# Supplementary material for: Canine distemper in Nepal's Annapurna Conservation Area – Implications of dog husbandry and human behaviour for wildlife disease
Source: PLoS One. 2019 Dec 5;14(12):e0220874. doi: 10.1371/journal.pone.0220874 (PMC6894829; doi:10.1371/journal.pone.0220874)
Supplement: S1 Table — Variables were clustered into two levels–household, and dog. Based on the published literature, aspects of the household, and dog level were included for assessment in the questionnaire. (PDF) [file pone.0220874.s001.pdf]

S1 Table

Level	Variable	Justification	Ref
Household	Site	The environmental in which a dog resides can influence its risk of exposure and susceptibility to disease.	(Larson and Schultz 2007, Sonne et al. 2007)
	Elevation	The distribution of human populations is inversely related to elevation. As domestic dogs are ubiquitously associated with human populations, it is meaningful to explore the relationship between the distribution of the human population and dog abundance across an elevation gradient. Elevation can also have effects on infectious disease and host physiology.	(Baker 1969, De Jong 1970, Kopec 1971, Cohen and Small 1998, Grover et al. 1998, Glaus et al. 2003a, Glaus et al. 2003b, Basnyat et al. 2009)
	Dogs per household	Evaluating dog population size is fundamental for disease management. Disease risk has been associated with the number of dogs per household.	(WHO 1988, Perry 1993, Haydon et al. 2002, Reithinger et al. 2003)
	Household income	Socio-economic status can influence ability to afford prophylactics before, during, and after exposure to a pathogen.	(Fung et al. 2014)
	People per household	The association between dog ownership and household size and income has consistently been reported in several studies.	(Franti et al. 1980, Wise and Yang 1992, Hsu et al. 2003, Westgarth et al. 2007, Knobel 2008)
Dog	Roaming behaviour	The level of restriction imposed on the movement of a dog can have ramifications in the context of infectious disease.	(Perry 1993, Hughes and Macdonald 2013, Kustritz 2014)
	Function	Dog function has implications on its behaviour and may affect its aggression toward other animals.	(Broom 2006, Gompper 2014)
	Sterilised status	Neutering dogs can have beneficial or adverse effects on its health.	(Villamil et al. 2009, Beauvais et al. 2012)
	Body condition score	Body condition is an indicator of health and can have ramifications for the frequency and kind of interactions that dogs have with wildlife. It can also suggest a dog's ability to stave off the deleterious effects of disease.	(Kealy et al. 2002, Silva-Rodriguez and Sieving 2011)
	Dog age<br>Dog sex	Sex and age can be strongly associated with an individual's susceptibility to infection. Sex and age ratios can affect the transfer and maintenance of pathogens.	(Grenfell and Dobson 1995, Thrusfield 2005, Real and Biek 2007)

© Ng *et al.*, 2019
