# Supplementary material for: Canine distemper in Nepal's Annapurna Conservation Area – Implications of dog husbandry and human behaviour for wildlife disease
Source: PLoS One. 2019 Dec 5;14(12):e0220874. doi: 10.1371/journal.pone.0220874 (PMC6894829; doi:10.1371/journal.pone.0220874)
Supplement: S2 Table — Numbers reflect reported livestock deaths associated with the corresponding predator. One rancher reported losing a total of 35 goats to a four different predators, but was unable to separate the number of livestock deaths associated with each predator. His report has been excluded from the table below. Also excluded from the table is the report by one rancher who lost more than 100 goats. The “Unknown” category describes reported livestock deaths associated with a predator attack of indiscernible identity. (PDF) [file pone.0220874.s002.pdf]

S2 Table

	Predator						
**Livestock**		Dog	Fox	Jackal	Snow leopard	Wolf	Unknown
	Bull						1
	Chauri				28	1	
	Chicken	1	2				4
	Cow				1		1
	Goat	3		2	3		1
	Horse				3		
	Yak				23		
