# Supplementary material for: Canine distemper in Nepal's Annapurna Conservation Area – Implications of dog husbandry and human behaviour for wildlife disease
Source: PLoS One. 2019 Dec 5;14(12):e0220874. doi: 10.1371/journal.pone.0220874 (PMC6894829; doi:10.1371/journal.pone.0220874)
Supplement: S3 Table — The table shows the S/P ratio that was derived from calculations as described in the methods, and the corresponding result. (PDF) [file pone.0220874.s003.pdf]

S3 Table

SN	S/P ratio	Result	SN	S/P ratio	Result
1	0.41	Positive	44	0.27048	Positive
2	0.67	Positive	45	0.47363	Positive
3	0.12	Negative	46	0.28844	Positive
4	0.34	Positive	47	0.27385	Positive
5	0.26	Positive	48	0.24916	Positive
6	0.22	Negative	49	0.3569	Positive
7	0.18	Negative	50	0.22783	Negative
8	0.36	Positive	51	0.79461	Positive
9	0.69	Positive	52	0.04938	Negative
10	0.15	Negative	53	0.27385	Positive
11	0.52	Positive	54	0.3569	Positive
12	0.16835	Negative	55	0.36588	Positive
13	0.27609	Positive	56	0.3367	Positive
14	0.36027	Positive	57	0.35578	Positive
15	0.58361	Positive	58	0.13356	Negative
16	0.55219	Positive	59	0.21886	Negative
17	0.37486	Positive	60	0.37823	Positive
18	0.37374	Positive	61	0.25028	Positive
19	0.29405	Positive	62	0.17396	Negative
20	0.35915	Positive	63	0.28956	Positive
21	0.17733	Negative	64	0.41639	Positive
22	0.15264	Negative	65	0.15488	Negative
23	0.55668	Positive	66	0.20651	Negative
24	0.3468	Positive	67	0.37935	Positive
25	0.14366	Negative	68	0.2413	Negative
26	0.31089	Positive	69	0.33221	Positive
27	0.15152	Negative	70	0.47699	Positive
28	0.46577	Positive	71	0.28058	Positive
29	0.15376	Negative	72	0.25926	Positive
30	0.39282	Positive	73	0.77104	Positive
31	0.48709	Positive	74	0.54097	Positive
32	0.50617	Positive	75	0.37149	Positive
33	0.16049	Negative	76	0.49944	Positive
34	0.36364	Positive	77	0.22559	Negative
35	0.65993	Positive	78	0.20314	Negative
36	0.40292	Positive	79	0.15937	Negative
37	0.31313	Positive	80	0.16835	Negative
38	0.2312	Negative	81	0.34343	Positive
39	0.40067	Positive	82	0.41077	Positive
40	0.33782	Positive	83	0.30303	Positive
41	0.23681	Negative	84	0.30752	Positive
42	0.45679	Positive	85	0.63749	Positive
43	0.38272	Positive			
