# Supplementary material for: Canine distemper in Nepal's Annapurna Conservation Area – Implications of dog husbandry and human behaviour for wildlife disease
Source: PLoS One. 2019 Dec 5;14(12):e0220874. doi: 10.1371/journal.pone.0220874 (PMC6894829; doi:10.1371/journal.pone.0220874)
Supplement: S1 Appendix — The complete questionnaire that was administered to residents in the study area. (PDF) [file pone.0220874.s004.pdf]

S1 Appendix

Interviewer Date (dd/mm)

Site[1] M, P, B, G, N, K GPS location

Name of interviewee (M/F)

Is interviewee the owner of the house (Y/N)

No. of people in the household? No. of children (<18years)

I.- QUESTIONS ABOUT OWNED DOG(S)

1. How many dogs do you have? ____________________________
   If no dogs, skip to PART II.
2. How many puppies (dogs < 3 mths) ________________________

3. Dog(s) details:

Name	Sex	Age[2]	Breed	Source[3]	Function[4]	Roaming[5]	kg	Sterilised
	M/F			N/BO/F/BN	G/He/P/H	N/A/S		Y/N
	M/F			N/BO/F/BN	G/He/P/H	N/A/S		Y/N
	M/F			N/BO/F/BN	G/He/P/H	N/A/S		Y/N
	M/F			N/BO/F/BN	G/He/P/H	N/A/S		Y/N

4. Do your neighbours' dog/s roam freely in the neighbourhood?*
   a. Always
   b. Sometimes (describe)__________________________________
   c. Never

5. Do you see free-roaming dogs of unknown/no owner in the neighbourhood?*
   a. Always
   b. Sometimes (describe)__________________________________
   c. Never

---

[1] m = Manang/Humre, p = Pisang, b = Bhraka, g = Ghyaru, n = Ngawal, k = Khangsar
[2] P = Pups (0–4 mo), j = Juveniles (5–12 mo), a = Adults (>12 mo)
[3] N: acquired from neighbours, BO: bought, F: found, BN: born at home
[4] G: guarding, He: herding, P: pet, H: hunting
[5] N: never, A: always, S: sometimes

6. How do you feed your dog(s) (Specify: kind of food and frequency)?

Kind of food	No. times/day

7. How do you dispose your waste?
   a. Open Dump
   b. Closed Dump
   c. Burning
   d. At a central dump (municipal)
   e. Other (specify)____

8. If your dog is a female fill in the table below (Fertility):

1. Name of female dog	2. **Total** no. of litters	3. Litter in last 12 mths?	4. If yes, give months	**Puppies from last 12 months**					
				5. Litter size	6.No. given away	7. Free-roaming (abandoned)	8a. No. died	8b. Cause of death	9.No. remaining?
		Y/N							

9. Do you de-worm your dogs? (Y/N) If 'Y', with what ___________ how often? _________

10. Have your dog(s) have been seen by a veterinary? (Y/N)
    If 'Y', go to Q11. If 'N', go to Q12.

11. How often have you visited the veterinary with your dog(s) in the past 12 months?_____

12. Vaccination status of your dog(s)

Name of dog	Vaccinated (Y/N)	Vaccine[6]	Date of vaccination (Month/Yr)

13. How many dogs died in the last 12 months_________________________?

[6]Rabies (R), CDV, CPV

14. For dogs that died complete the table below:

Name	Age	Sex	Date	Causes			
				Killed by human (describe)	Old age	Disease	Other (describe)
		M/F					
		M/F					
		M/F					
		M/F					

15. How many dogs got sick in the past 12 months ________________________?

16. Regarding sick animals. Which clinical signs did the dogs that died/get sick show?

CLINICAL SIGN	NAME OF DOG				
Coughing					
Sneezing					
Nasal Discharge					
Blindness					
Blue eyes					
Lacrimation watery eyes					
Anorexia wants to eat but difficulty picking up, chewing, or swallowing food, decease in appetite					
Emaciation loss of fat, ribs showing					
Salivation					
Vomiting					
Diarrhoea					
Vocalization incessant barking/howling – if yes, what time of the day does vocalisation occur?	Time of day	Time of day	Time of day	Time of day	Time of day
Change of behaviour					
Ataxia loss of coordination, walks funny					
Convulsions sudden, ***violent***, uncontrolled muscle movement					
Muscle twitching					
Paralysis loss of muscle function, whole or partial					
Coma loss of consciousness					
Death					
Others					

II.- ATTITUDES REGARDING COMMUNITY DOGS

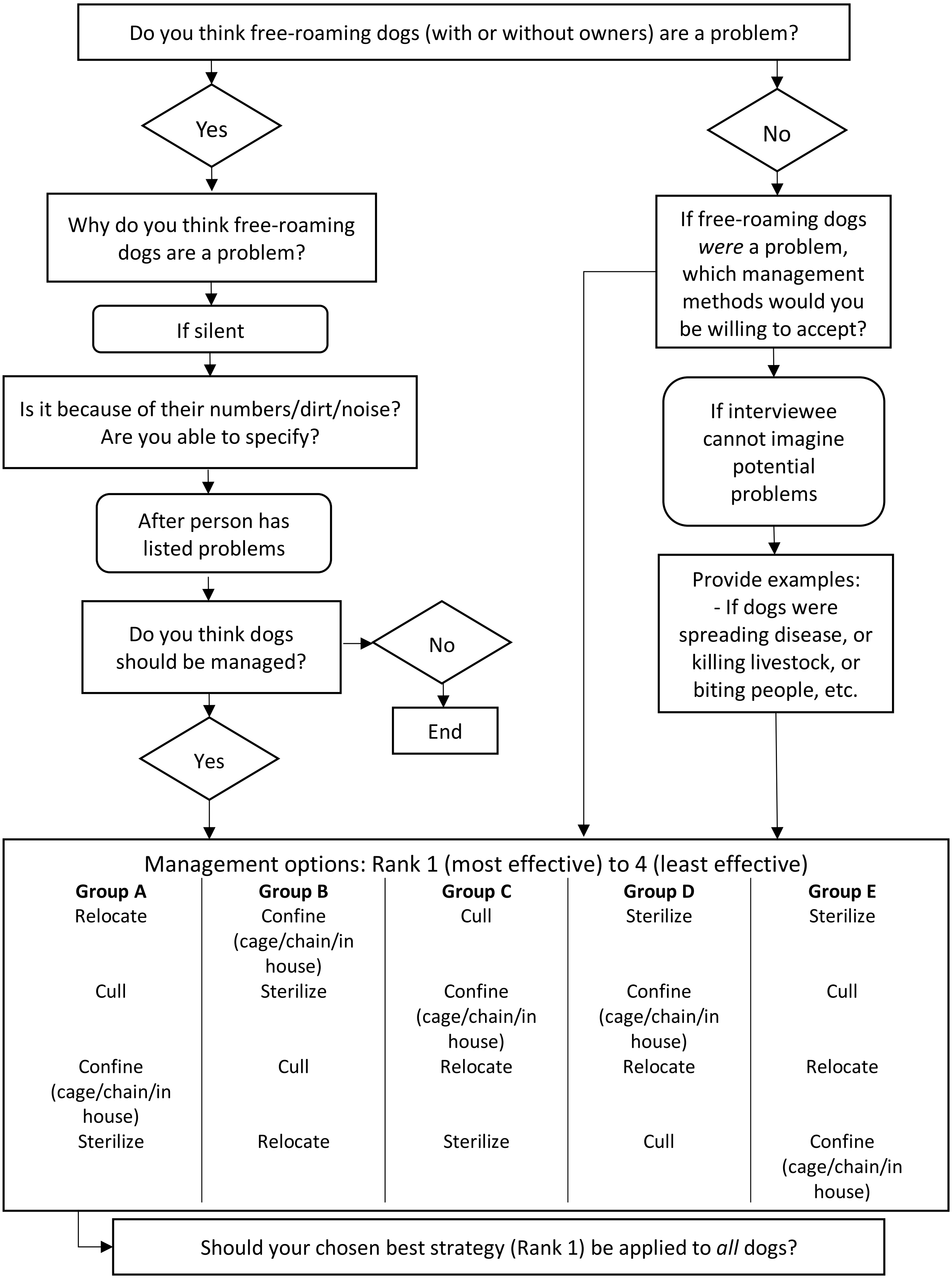

III. QUESTIONS REGARDING LIVESTOCK AND WILDLIFE

17. Which of these wild animals exist in **this area**? (Showing pictures) 1 2 3 4 5 6

18. Have you seen any of these animals **close to your house or your animals**? Y/N.
    If 'Y', which animal/s? 1 2 3 4 5 6

19. Which of the animals identified in Q18 come close your house **more often**?
    1 2 3 4 5 6

20. What other animals (species and number) do you have in your home?

Cat	Goat	Sheep	Cow	Horse	Mule	Jyupa	Yak

21. Have your livestock been attacked by **wild** carnivores, Y/N
    If 'Y', follow to Q22. If 'N', go to Q23.

22. Please point out kind of livestock and number of animals killed by these attacks in the last year and the wild animal that likely caused them.

Kind of livestock	No. killed	Wild predator

    a. Was the attack witnessed? (Y/N), If 'Y' go to Q23.
    b. If 'N', how was the species of predator determined?
    ____

23. Have you seen your dog(s) in contact with **wild animals**? Y/N
    If 'Y', go to Q24-25. If 'N', go to Q26.

24. With what animals have you seen your dog(s) in contact with?
    ____

25. What were you or your dog doing when the encounter happened? Season? Weather?
    ____
    ____
    ____

26. Of the animals in the pictures, have you seen any with **signs of diseases**? Y/N
    If 'Y', please describe:

Animal (1-6)	Place	Date or year /Season	Symptoms (ref. Q16)	Other details

IV. Questions regarding socioeconomic condition

27. Household condition
    a. Own
    b. Rented
    c. Family house
    d. Others (Specify)

28. Education of household owner
    a. No formal education
    b. Primary (Grade 1-5)
    c. Lower Secondary (Grade 6-8)
    d. Secondary (Grade 9-10)
    e. Higher Secondary (Grade 11-12)

END OF INTERVIEW

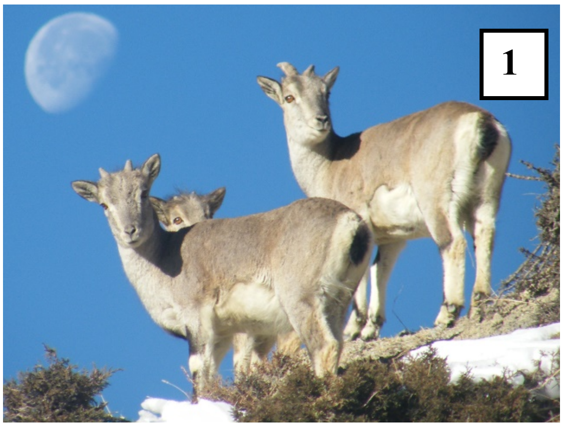
1

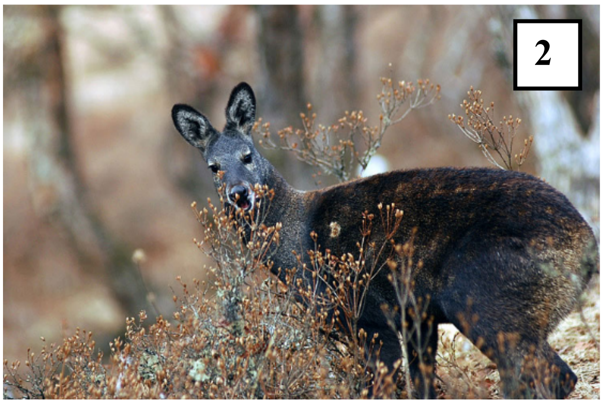
2

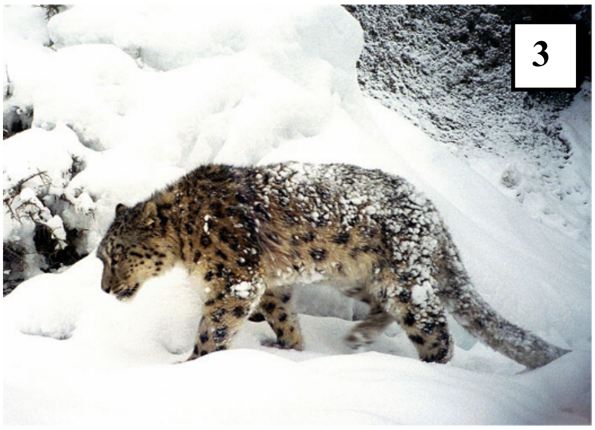
3

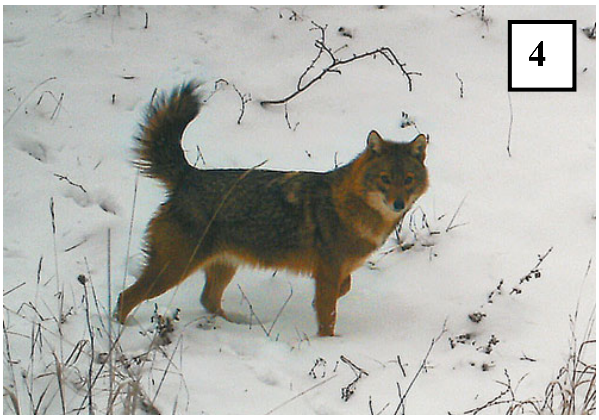
4

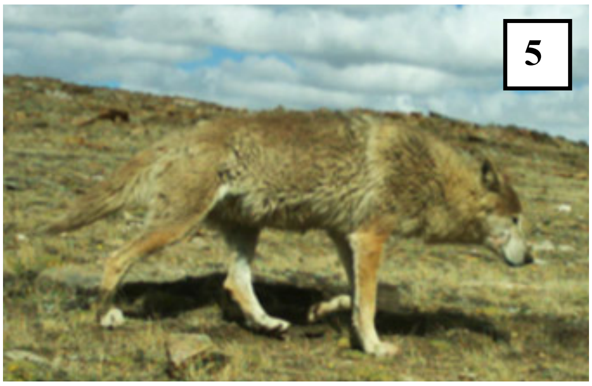
5

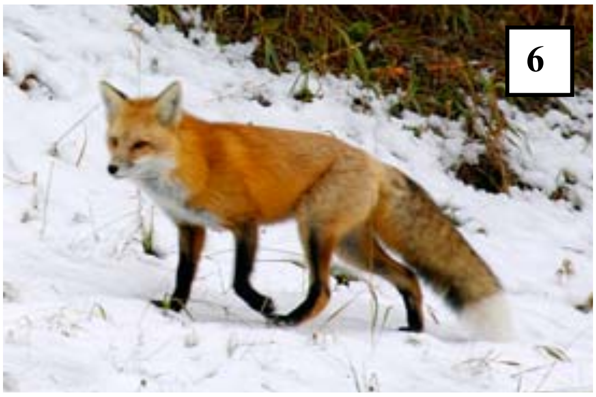
6
