# Supplementary material for: Canine distemper in Nepal's Annapurna Conservation Area – Implications of dog husbandry and human behaviour for wildlife disease
Source: PLoS One. 2019 Dec 5;14(12):e0220874. doi: 10.1371/journal.pone.0220874 (PMC6894829; doi:10.1371/journal.pone.0220874)
Supplement: S2 Appendix — The data sheet that was used to describe dog-level variables. Skin, body condition and BCS were ascertained by the same veterinarian throughout the study. Age was also ascertained by the veterinarian if such information was not available from the dogs’ owner. (PDF) [file pone.0220874.s005.pdf]

S2 Appendix

Researcher: Date: ID:

Owners name: Dogs name:

Location Sex (M/F) Sterilised (Y/N)

Tube no. Vaccination: Rabies/CDV/CPV

Photo no. Function: **G**uarding/**H**erding/**P**et/Hu**n**ting

Dog's Age: Pup (3–5 mo), Juv (5–12 mo) Adults (>12 mo)

Breed:

Weight (kg) Length (mm)

Source[7]: N / Bt / F / Bn

Roaming: Never / Always / Sometimes

Skin Condition: none / < 20% / > 20% 9-point BCS[8]: /9

Body condition notes:

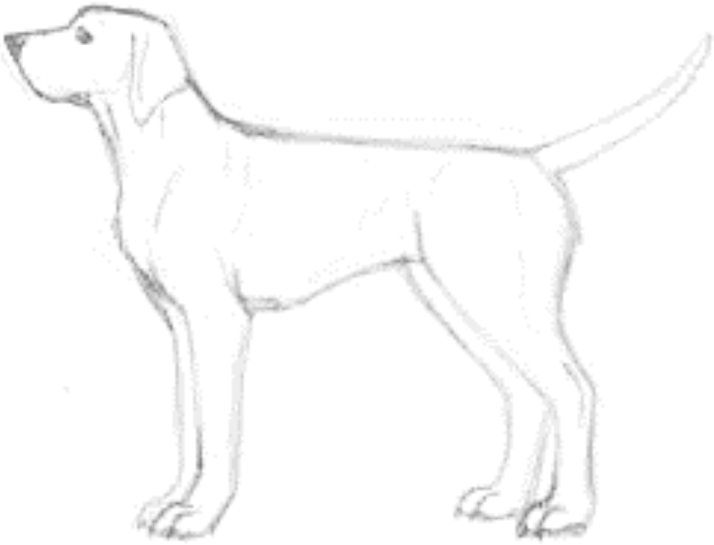

Comments: ______________________________

____

____

____

[7] N: acquired from neighbours, Bt: bought, F: found, Bn: born at home
[8] 1 (very thin) 5 (Ideal) 9 (obese)

© Ng *et al.*, 2019
