# Supplementary figures and images for: Canine distemper in Nepal's Annapurna Conservation Area – Implications of dog husbandry and human behaviour for wildlife disease
Source: PLoS One. 2019 Dec 5;14(12):e0220874. doi: 10.1371/journal.pone.0220874 (PMC6894829; doi:10.1371/journal.pone.0220874)

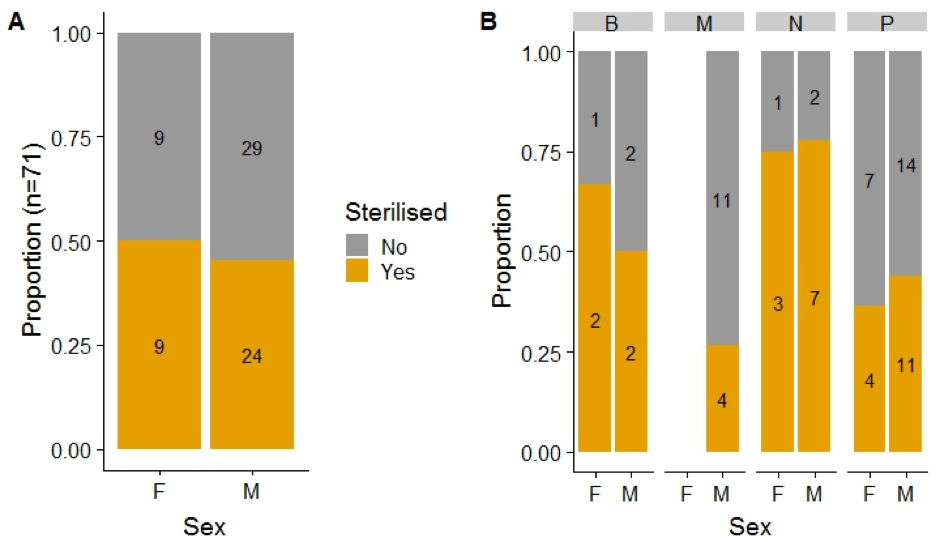

Supplement: S1 Fig — Sex and sterilised status of owned dogs in (A) study area, and (B) within each village cluster. B: Bhraka (n = 7), M: Manang (n = 15), N: Ngawal (n = 13), P: Pisang (n = 36). Numbers in bars represent number of observations. Sterilised status across the study area was relatively evenly distributed between males and females. However, note the completely male-biased population in Manang and the low rate of sterilisation. (PNG) [file pone.0220874.s006.png]

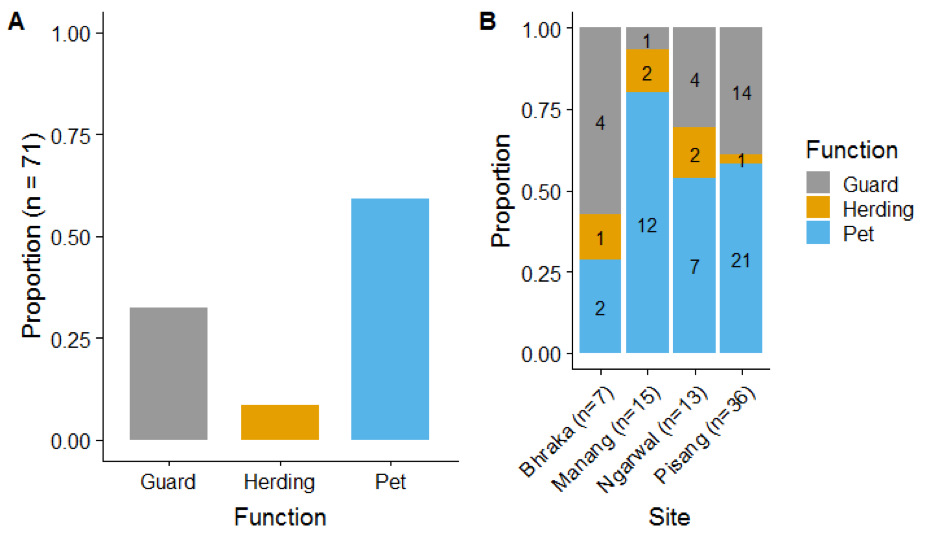

Supplement: S2 Fig — Function of owned dogs as described by their owners in (A) study area, and (B) within each village. Numbers in bars represent number of observations. Manang and Pisang had the greatest proportion of pet dogs, while Bhraka had the greatest proportion of dogs kept for utility. (PNG) [file pone.0220874.s007.png]

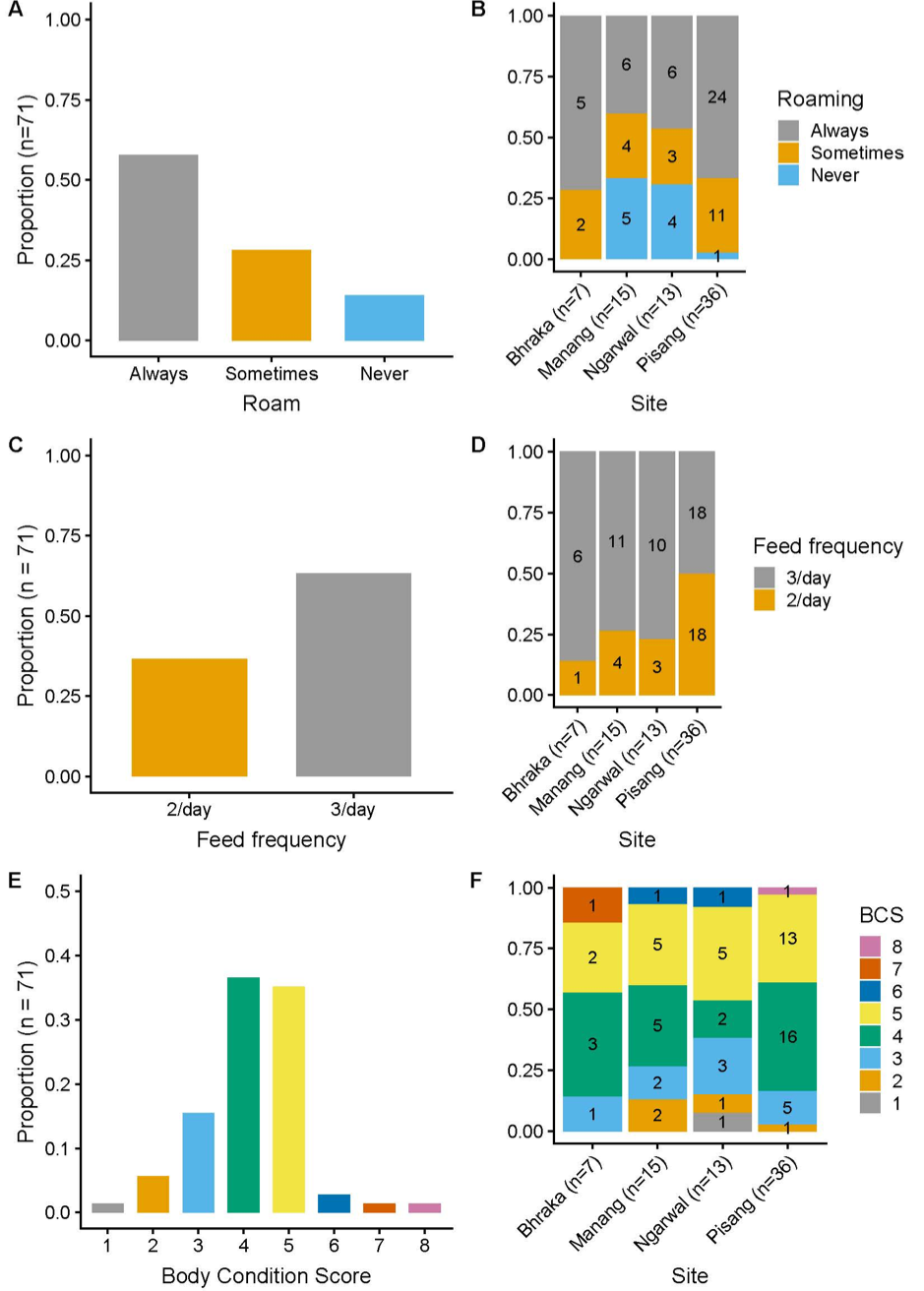

Supplement: S3 Fig — Roaming restrictions (A-B), feed frequency (C-D) and body condition of dogs (E-F) in the study area (A,C,E) and within each village cluster (B,D,F). More than half of all dogs with owners were allowed to roam freely (A). Pisang and Bhraka had the largest proportion of free-roaming dogs (B). Most owned dogs were fed three times a day (C), except in Pisang where half the dog population was fed twice a day. Most dogs had an optimal BCS (E). Bhraka and Pisang had the largest proportion of dogs with ideal BCS, while Ngarwal had the largest proportion with a BCS <4. No dogs were scored 9. Numbers within the bars represent the number of observations. (PNG) [file pone.0220874.s008.png]

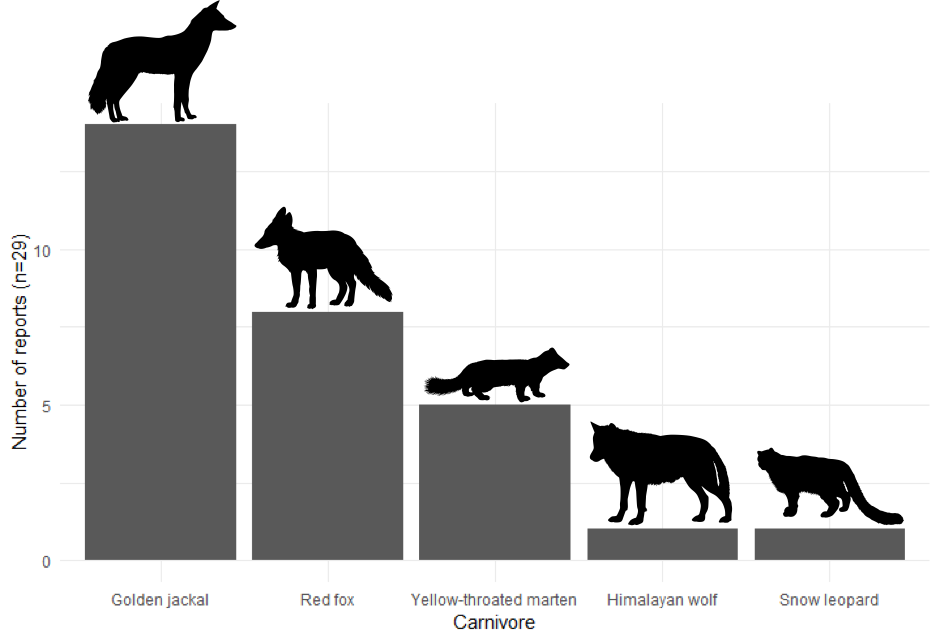

Supplement: S4 Fig — Golden jackal: Canis aureus, and red fox: Vulpes vulpes, were the two most commonly sighted predators. (TIF) [file pone.0220874.s009.tif]
