# Supplementary material for: Canine distemper in Nepal's Annapurna Conservation Area – Implications of dog husbandry and human behaviour for wildlife disease
Source: PLoS One. 2019 Dec 5;14(12):e0220874. doi: 10.1371/journal.pone.0220874 (PMC6894829; doi:10.1371/journal.pone.0220874)
Supplement: S1 File — The complete model outputs from the MuMIn package that was used to run all possible model combinations of predictor variables for dogs per household, and all single and pairwise predictor variable combinations for CDV exposure. (PDF) [file pone.0220874.s010.pdf]

S1 File

Term codes for predicting CDV seroprevalence

Body condition (bcs)	Dog age (dogage)	Dog sex (dogsex)	Function (func)	Household income (income)	People per household (nhouse)	Roaming (roam)	Site (site)	Sterilised status (ster)
1	2	3	4	5	6	7	8	9

Component models

Model terms	df	logLik	AICc	delta	weight
2+8	6	-24.35	62.34	0	0.29
8	4	-27.52	63.79	1.45	0.14
5+8	5	-26.41	63.97	1.63	0.13
4+8	6	-25.21	64.07	1.73	0.12
8+9	5	-26.53	64.22	1.87	0.11
1+8	5	-27.15	65.45	3.11	0.06
3+8	5	-27.5	66.15	3.81	0.04
6+8	5	-27.5	66.16	3.81	0.04
7+8	6	-27.45	68.55	6.21	0.01
2	3	-31.82	70.09	7.75	0.01
9	2	-33.33	70.88	8.53	0
2+6	4	-31.11	70.98	8.64	0
1+9	3	-32.34	71.12	8.77	0
2+3	4	-31.18	71.12	8.78	0
2+4	5	-30.03	71.21	8.87	0
2+5	4	-31.28	71.31	8.96	0
5+9	3	-32.44	71.33	8.98	0
3+9	3	-32.44	71.33	8.98	0
1+2	4	-31.37	71.49	9.15	0
6+9	3	-32.81	72.06	9.71	0
2+9	4	-31.66	72.07	9.72	0
3	2	-34.3	72.83	10.48	0
5	2	-34.34	72.9	10.56	0
4+9	4	-32.16	73.08	10.73	0
6	2	-34.43	73.08	10.74	0
2+7	5	-31.13	73.41	11.06	0
3+5	3	-33.59	73.63	11.29	0
1	2	-34.85	73.92	11.58	0
5+6	3	-33.79	74.03	11.69	0
3+6	3	-33.92	74.28	11.94	0
1+3	3	-33.99	74.42	12.07	0
4	3	-34.01	74.46	12.12	0
4+5	4	-32.91	74.57	12.23	0
1+6	3	-34.15	74.74	12.4	0
4+6	4	-33.02	74.79	12.45	0

1+5	3	-34.22	74.88	12.54	0
7+9	4	-33.16	75.07	12.73	0
3+4	4	-33.21	75.18	12.84	0
7	3	-34.75	75.95	13.61	0
1+4	4	-33.85	76.46	14.12	0
5+7	4	-33.87	76.49	14.14	0
3+7	4	-34.04	76.84	14.5	0
1+7	4	-34.15	77.06	14.71	0
6+7	4	-34.17	77.09	14.74	0
4+7	5	-33.52	78.19	15.85	0

Model averaged coefficients:
(full average)

	Estimate	Std. Error	Adjusted SE	z value	Pr(>|z|)
(Intercept)	6.46E+00	2.68E+03	2.74E+0<br>3	0.00<br>2	0.998
dogage2	-5.00E+00	2.68E+03	2.74E+03	0.002	0.999
dogage3	-5.68E+00	2.68E+03	2.74E+03	0.002	0.998
siteM	1.71E+01	1.94E+03	1.99E+03	0.009	0.993
siteN	-4.03E-01	1.13E+00	1.15E+00	0.35	0.726
siteP	-5.97E-01	9.64E-01	9.86E-01	0.605	0.545
income	3.34E-05	1.14E-04	1.15E-04	0.291	0.771
funche	-2.74E-01	8.75E-01	8.82E-01	0.31	0.756
funcp	-1.74E-01	5.36E-01	5.40E-01	0.323	0.747
ster1	-1.34E-01	4.32E-01	4.35E-01	0.308	0.758
bcs	3.47E-02	1.99E-01	2.01E-01	0.172	0.863
dogsex1	1.51E-02	1.81E-01	1.85E-01	0.082	0.935
nhouse	1.97E-04	4.72E-02	4.82E-02	0.004	0.997
roam2	-3.90E-03	1.51E-01	1.55E-01	0.025	0.98
roam3	-6.99E-03	1.55E-01	1.58E-01	0.044	0.965

(conditional average)

	Estimate	Std. Error	Adjusted SE	z value	Pr(>|z|)
(Intercept)	6.46E+00	2.68E+03	2.74E+03	0.002	0.9981
dogage2	-1.60E+01	4.79E+03	4.90E+03	0.003	0.9974
dogage3	-1.82E+01	4.79E+03	4.90E+03	0.004	0.997
siteM	1.82E+01	2.00E+03	2.05E+03	0.009	0.9929
siteN	-4.27E-01	1.16E+00	1.18E+00	0.362	0.7174
siteP	-6.33E-01	9.81E-01	1.00E+00	0.63	0.5284
income	2.42E-04	2.08E-04	2.13E-04	1.14	0.2543
funche	-2.14E+00	1.41E+00	1.45E+00	1.482	0.1384
funcp	-1.36E+00	7.94E-01	8.12E-01	1.678	0.0934 .
ster1	-1.01E+00	7.21E-01	7.37E-01	1.373	0.1697
bcs	4.95E-01	5.79E-01	5.92E-01	0.837	0.4027
dogsex1	2.79E-01	7.32E-01	7.47E-01	0.374	0.7082
nhouse	3.74E-03	2.06E-01	2.10E-01	0.018	0.9858
roam2	-2.49E-01	1.18E+00	1.21E+00	0.206	0.8371

roam3	-4.45E-01	1.15E+00	1.18E+00	0.378	0.7056

Relative variable importance:

	site	dogage	income	ster	func	bcs	dogsex	nhouse	roam
Importance:	0.94	0.31	0.14	0.13	0.13	0.07	0.05	0.05	0.02
N containing models:	9	9	9	9	9	9	9	9	9

Term codes for predicting dog abundance per household

Elevation (elv)	Function (func)	Household Income (income)	People per household (nhouse)	Site (site)
1	2	3	4	5

Component models:

Model terms:	df	logLik	AICc	delta	weight
3	2	-68.99	142.21	0	0.21
(Null)	1	-70.18	142.42	0.22	0.18
3+4	3	-68.8	144.04	1.83	0.08
4	2	-70.03	144.27	2.06	0.07
1+3	3	-68.99	144.43	2.22	0.07
1	2	-70.15	144.53	2.32	0.06
2	3	-69.06	144.56	2.35	0.06
2+3	4	-68.39	145.54	3.33	0.04
1+3+4	4	-68.8	146.35	4.14	0.03
1+4	3	-70.02	146.48	4.27	0.02
2+4	4	-68.89	146.53	4.32	0.02
1+2	4	-69.01	146.78	4.58	0.02
1+5	5	-68.01	147.18	4.97	0.02
5	4	-69.22	147.19	4.98	0.02
2+3+4	5	-68.2	147.56	5.35	0.01
3+5	5	-68.32	147.8	5.59	0.01
1+2+3	5	-68.38	147.92	5.71	0.01
1+3+5	6	-67.32	148.29	6.08	0.01
1+2+4	5	-68.86	148.88	6.67	0.01
2+5	6	-67.91	149.47	7.26	0.01
1+4+5	6	-67.95	149.54	7.33	0.01
4+5	5	-69.2	149.56	7.36	0.01
1+2+5	7	-66.89	150.01	7.8	0
1+2+3+4	6	-68.2	150.04	7.84	0
3+4+5	6	-68.27	150.2	7.99	0
1+3+4+5	7	-67.3	150.85	8.64	0
2+3+5	7	-67.58	151.4	9.2	0
2+4+5	7	-67.86	151.97	9.76	0
1+2+3+5	8	-66.67	152.29	10.08	0
1+2+4+5	8	-66.87	152.68	10.47	0
2+3+4+5	8	-67.51	153.96	11.75	0
1+2+3+4+5	9	-66.67	155.09	12.88	0

Model-averaged coefficients:

(full average)

	Estimate	Std. Error	Adjusted SE	z value	Pr(>|z|)
(Intercept)	-1.04E-01	2.83E+00	2.86E+00	0.036	0.971
income	3.15E-05	4.38E-05	4.42E-05	0.712	0.477
nhouse	-8.49E-03	3.39E-02	3.45E-02	0.246	0.806
elv	9.95E-05	8.05E-04	8.13E-04	0.122	0.903
funche	6.33E-02	2.28E-01	2.31E-01	0.274	0.784
funcp	-4.13E-02	1.42E-01	1.44E-01	0.286	0.775
siteM	-4.94E-03	1.53E-01	1.56E-01	0.032	0.975
siteN	5.65E-04	1.91E-01	1.95E-01	0.003	0.998
siteP	5.41E-02	2.31E-01	2.33E-01	0.232	0.816

(conditional average)

	Estimate	Std. Error	Adjusted SE	z value	Pr(>|z|)
(Intercept)	-1.04E-01	2.83E+00	2.86E+00	0.036	0.971
income	6.52E-05	4.21E-05	4.30E-05	1.515	0.13
nhouse	-3.09E-02	5.91E-02	6.04E-02	0.512	0.609
elv	3.72E-04	1.52E-03	1.54E-03	0.242	0.809
funche	3.16E-01	4.23E-01	4.32E-01	0.732	0.464
funcp	-2.06E-01	2.58E-01	2.64E-01	0.78	0.436
siteM	-5.50E-02	5.06E-01	5.17E-01	0.106	0.915
siteN	6.29E-03	6.39E-01	6.50E-01	0.01	0.992
siteP	6.02E-01	5.14E-01	5.23E-01	1.151	0.25

Relative variable importance:

income	nhouse	elv	func	site
0.48	0.27	0.27	0.2	0.09
16	16	16	16	16
